# Pathways to Science and Engineering Bachelor’s Degrees for Men and Women

**DOI:** 10.15195/v1.a4

**Published:** 2014-02-18

**Authors:** Joscha Legewie, Thomas A. DiPrete

**Affiliations:** a Wissenschaftszentrum Berlin für Sozialforschung; b Department of Sociology, Columbia University

**Keywords:** gender, education, STEM fields

## Abstract

Despite the striking reversal of the gender gap in educational attainment and the near–gender parity in math performance, women pursue science and engineering (S/E) degrees at much lower rates than their male peers do. Current efforts to increase the number of women in these fields focus on different life-course periods but lack a clear understanding of the importance of these periods and how orientations toward S/E fields develop over time. In this article, we examine the gendered pathways to a S/E bachelor’s degree from middle school to high school and college based on a representative sample from the 1973 to 1974 birth cohort. Using a counterfactual decomposition analysis, we determine the relative importance of these different life-course periods and thereby inform the direction of future research and policy. Our findings confirm previous research that highlights the importance of early encouragement for gender differences in S/E degrees, but our findings also attest to the high school years as a decisive period for the gender gap, while challenging the focus on college in research and policy. Indeed, if female high school seniors had the same orientation toward and preparation for S/E fields as their male peers, the gender gap in S/E degrees would be closed by as much as 82 percent.

Despite the striking reversal of the gender gap in educational attainment (Buchmann and DiPrete 2006; Legewie and DiPrete 2009) and the near–gender parity in math performance (Hyde et al. 2008), women pursue science and engineering (S/E) degrees at much lower rates than their male peers do. This persisting gender gap is relevant not only for gender equality but also for the supply of qualified labor in science-oriented jobs—a linchpin for the future of the U.S. economy in an increasingly competitive global environment.

Now that women graduate from high school with equal preparation for a science career as men (DiPrete and Buchmann 2013), many researchers have shifted to concentrate on college and beyond as the decisive life-course period for explaining the S/E gender gap. Along these lines, a 2005 contribution to the *Policy Forum in Science* magazine titled “More Women in Science” highlights work–family balance, climate, and unconscious bias in higher education as the main reasons why women exit a science career (Handelsman, et al. 2005), encapsulating the focus of today’s research (Cech, et al. 2011; Ceci and Williams 2011; Moss-Racusin et al. 2012). At the same time, research finds that boys and girls develop different occupational orientations during early childhood that are highly consequential for later career choices (Tai et al. 2006). In this line, Morgan, Gelbgiser, and Weeden (2013) have shown that gender-specific occupational orientations as of the senior year of high school explain much of the gender difference in college majors. Many also highlight the importance of the number and quality of teachers in primary and secondary schools (National Research Council 2005) as well as early inputs in general (Heckman 2006).

Despite this focus on different life-course periods, much uncertainty remains as to when a stable gender gap in orientation toward S/E fields appears and the importance of these different life-course periods in contributing to the divergence. Some researchers have studied the later gendered pathways to a degree in S/E fields, starting at the end of high school and following students through to the completion of college (Xie and Shauman 2005; Morgan et al. 2013). Yet no previous study to our knowledge has examined the development of science orientation over a more extended period, beginning with middle school and then tracking students through to high school and college graduation. However, understanding the importance of these different life-course periods and the various pathways taken by students is essential for an effective deployment of research and policy resources.

Here we follow the emergence of science expectations based on three snapshots of S/E orientation at key points in time—middle school, end of high school, and graduation from college—and use a counterfactual decomposition analysis to determine the contribution of these three life-course periods to the persisting gender gap in S/E bachelor’s degree attainment.

## Data and Methods

To examine the role of different life-course periods, we use a representative sample of 10,230 eighth-grade students from the 1973 to 1974 birth cohort who were followed over time in the National Education Longitudinal Study (NELS) from 1988 to 2000. NELS is a nationally representative sample of about 25,000 eighth-grade students who were first surveyed in spring 1988. Subsamples of these students were resurveyed in 1990, 1992, 1994, and 2000 so that the students were followed over time as they graduated from high school and entered the labor force or pursued postsecondary degrees. The fact that the survey begins with students in eighth grade makes NELS uniquely suited for our purpose because it allows us to track orientations toward S/E fields over an extended period (the more recent ELS, for example, first surveyed students in high school). We restrict the NELS 1988 to 2000 sample to students who participated in the 8th- and 12th-grade surveys, and the 2000 follow-up, and exclude high school dropouts. The size of this restricted sample is 10,230. Out of these cases, 3,140 (30.7 percent) had missing information on at least one of the variables (mostly, 12th-grade test scores). We use multiple imputations based on the chained-equations approach to recover missing values (for details, see Appendix A in the supplement).

Using the panel structure of the data set, we decompose the probability that an individual graduates from college with a S/E bachelor’s degree into different pathways. The pathways are defined by transition rates between S/E orientations at three stages of the educational career, and we determine the relative importance of these three stages using a counterfactual decomposition analysis. The first stage is defined by expectations for a S/E career in eighth grade, as measured by the survey question “What kind of work do you expect to be doing when you are 30 years old?” The second stage is based on concrete plans to major in a S/E field in college at the end of high school (12th grade), measured by the survey question “Indicate the field that comes closest to what you would most like to study if you go to school.” These 8th- and 12th-grade variables are supplemented by measures for math and science performance and student interest to comprehensively capture pre–high school and high school gender differences in S/E orientation and preparation. Finally, the third stage is defined by whether a student has graduated from a four-year college with a S/E bachelor’s degree. See Appendix A for details about the data set and the coding of variables.

## Pathways to a Science and Engineering Bachelor’s Degree

**Figure 1:**
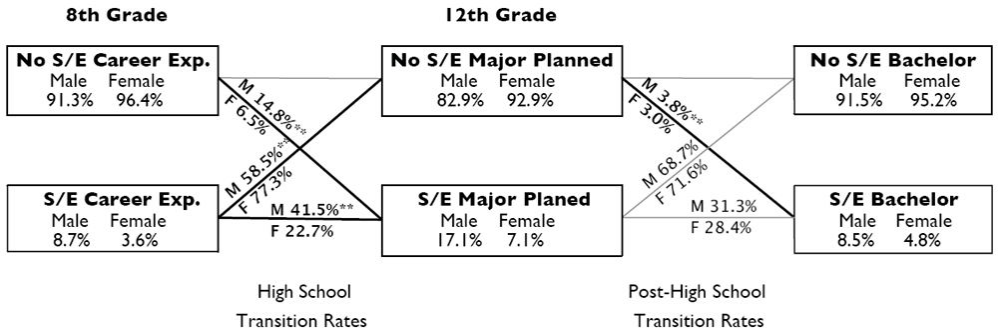
Pathways to a science and engineering BA degree (*p < 0.05; **p < 0.01).

Figure 1 shows a substantial gender gap in S/E career expectations in 8th grade. Boys are more than twice as likely as girls to expect to work in S/E in middle school (8.7 percent compared to 3.6 percent). These early science expectations both predict 12th-grade intentions to study science and increase the likelihood that students will earn a S/E bachelor’s degree. Specifically, boys and girls who expected to work in science-related careers in eighth grade were 3.8 and 3.3 times more likely to earn a S/E bachelor’s degree than students without such expectations, respectively. These findings highlight the importance of early encouragement for later science careers (Tai et al. 2006).

During the high school years, boys are both more likely to transition from being interested in pursuing a S/E career to planning to major in S/E upon graduation and to develop such plans without prior expectations of a S/E career (41.5 percent compared to 22.7 percent and 14.8 percent compared to 6.5 percent, respectively). After high school, however, boys and girls are equally likely to follow their plan to study S/E in college. In other words, once high school seniors have developed concrete plans to major in S/E, boys and girls are equally likely to pursue this plan and to graduate from college with a S/E bachelor’s degree (31.3 percent compared to 28.4 percent; the difference is not statistically significant). The proportion of students who abandon plans (exit rate) to pursue a S/E major during the transitions from middle school to high school to college reveal the same gender pattern. The gender gap in entry, however, remains substantial, even in the post–high school period. These findings demonstrate that boys are more likely to be recruited for S/E fields both during the high school years and following high school graduation. Thus the results in Figure 1 show pronounced (and highly significant) gender differences in the rate of entry into the science track based both on high school and post–high school transitions. Overall, these gender differences observed in transition pathways contribute to a substantial gender gap in S/E bachelor’s degree attainment by the end of college: only 4.8 percent of women in the 1973 to 1974 birth cohort obtained a S/E bachelor’s degree, as compared with 8.5 percent of men.

## Importance of Life-Course Periods

We use two counterfactual, Blinder–Oaxaca decomposition analyses to determine the contribution of the different life-course periods to the overall gender gap. These decompositions allow us to divide the gender gap in S/E bachelor’s degree attainment into two parts: the portion explained by gender differences in 8th- and 12th-grade characteristics and the unexplained portion that measures the role of post–high school choices and transitions (Yun 2004; Jann 2008). Our variables include S/E career expectations in 8th grade; planned college major in 12th grade; and math, science, and reading performance and math and science interest in 8th and 12th grades. By assigning women the characteristics of men (and, equivalently, men the characteristics of women), the decompositions simulate two counterfactual scenarios: how would the gender gap in S/E degrees change if women had the same orientation toward and preparation for S/E in middle school (decomposition 1) and at the end of high school (decomposition 2)? (See Appendix B in the supplement for details.)

Table 1 presents the results from these counterfactual decompositions. The results for the first decomposition in panel A indicate that the gender gap would be reduced by about 36 percent if women had the same orientation toward and preparation for S/E in eighth grade as men, whereas choices and transitions after middle school account for about 64 percent of the gap (the second term also includes unobserved factors; see Appendix B for details). The results from the corresponding decomposition using women’s distribution as the baseline are presented in Table B1. They show a similar reduction of about 29 percent if men had the same orientation toward and preparation for S/E in eighth grade as women, with 71 percent attributable to transitions after middle school.^1^ These results indicate that pre–high school gender differences play an important role and account for about one-third of the gender gap in S/E bachelor’s degree attainment.

The second decomposition, reported in panel B of Tables 1 and B1, includes additional variables that comprehensively capture the orientation toward and preparation for S/E at the end of high school. Together, 8th- and 12th-grade characteristics account for as much as 82 percent of the gender gap in S/E bachelor’s degree attainment (or 87 percent with women as the reference). Tables 1 and B1 also report the findings from the detailed decomposition, which allows us to distinguish between personal orientation regarding both career expectations and planned major as well as preparation in terms of performance. The results of this detailed decomposition are sensitive to the ordering of the variables. These ordering decisions reflect different causal models of educational decisions. Whereas the causal order of the 8th- and 12th-grade variables is determined by time, the causal order of the three variable sets is not clearly defined (8th-grade career expectations and 12th-grade major plans, math and science interest, and school performance). One possibility is that students first develop interests in math and science and then base their career expectations and efforts in school on these interests. Alternatively, parents might influence students’ career expectations, which in turn determines their interests and efforts. Finally, the performance of students might be decisive when the students themselves, parents, or teachers base further decisions on these abilities. As Morgan et al. (2013:999f) discuss, these three perspectives are all based on different strands of the literature. They are plausible, and they may operate jointly. Hence it is impossible to determine the causal ordering of the different factors with observed data. Instead, we present a range of results indicated by the notation [*x, y*], which corresponds to the minimum and maximum contributions from the range of possible causal orderings.

The results show that concrete study plans as of 12th grade account for a substantial proportion of the gender gap regardless of the assumed causal model. In particular, the estimates range from 41 percent to 68 percent with male as a reference and from 38 percent to 58 percent with female as a reference. This finding resembles Morgan et al.’s (2013) result for occupational plans. The contributions of the other two factors, however, largely depend on the ordering of the variables and therefore the implied causal model. Nonetheless, high school performance explains a sizable share of the gender gap ranging from up to 40 percent to about 6 percent. This contribution of performance is relatively large compared to other recent studies (Riegle-Crumb et al. 2012). Looking at the differences between students who actually graduate from four-year college with a bachelor’s degree, which is comparable to the sample restriction in other studies (Riegle-Crumb et al. 2012), substantially reduces this share and makes the two studies comparable (see Appendix C in the supplement).

Overall, these findings reaffirm previous research that highlights the importance of early encouragement (Tai et al. 2006) but also point to the high school years as the decisive period for the gender gap. At the same time, the results downplay the role of post–high school transitions and choices both outside and inside of college, which challenges the focus on college in much research and policy. Indeed, the gender gap would be reduced by as much as 82 percent (88 percent) if women (men) had the same S/E plans and the same preparation as men (women) at the end of high school. In contrast, post–high school transitions and choices only account for about 18 percent (12 percent), which is an upper-bound estimate, considering that unobserved factors are subsumed in this term.

We conducted the same analysis for different S/E subfields and for the subset of students who graduate from college with a four-year bachelor’s degree (see Appendix C). The results closely resemble the findings reported here. They indicate that the gender gap would be reduced by as much as 72.5 percent for physical science and engineering—the fields with the largest gender gaps—if women had the same orientation and preparation for these fields as men at the end of high school. Restricting our focus to the differences between those who actually obtain a four-year bachelor’s degree similarly indicates a reduction of 70.1 percent.

## Conclusion

Examining the pathways that lead to a bachelor’s degree in S/E fields reveals important insights about the role of different life-course periods in contributing to that endpoint, insights that partly challenge the current focus of research and public policy. On one hand, our findings confirm previous research that highlights the importance of early encouragement for the overall number of students with a S/E degree (Tai et al. 2006). Boys and girls with an orientation toward science-related careers in eighth grade are far more likely to pursue and obtain a S/E bachelor’s degree. Overall, the gender gap would be reduced by as much as 36 percent if women had the same eighth-grade characteristics as men. On the other hand, our counterfactual decompositions challenge the focus on the college years to close the gender gap in S/E fields. Instead, the persisting gender difference is largely a consequence of precollege orientation toward and preparation for S/E fields. In particular, if boys and girls had similar orientations toward S/E fields at the end of high school, the gender gap would be reduced by as much as 82 percent. These findings suggest that early encouragement and intervention during the high school years is an effective way to increase the overall number of students with S/E bachelor’s degrees and to close the gender gap. In turn, we also determine that research and policy efforts that focus on the post–high school period only target 18 percent or less of the gender gap in S/E bachelor’s degree attainment. Similar to recent research (Xie and Killewald 2012), these findings question the popularity of the notion that women are steered away from science during college—the leaky pipeline—as an explanation of the persisting gender gap. Instead, the high school years play a particularly important role. Along these lines, previous research has demonstrated the importance of the school context for gender differences in test scores (Legewie and DiPrete 2012) and interest in S/E fields (Legewie and DiPrete 2014). Some existing policy interventions have focused on high school students and shown success in promoting a S/E orientation among girls (Eisenhart 2008). Although such interventions have to withstand the serious scrutiny of experimental field trials, the evidence presented in this article encourages researchers and policy makers alike to take seriously the potential impact of high school interventions on the S/E orientations of female students.

## Supplementary Material

Supplement

## Figures and Tables

**Table 1:** Comparison of the Observed Gender Gap in Science and Engineering Degrees with the Gap under Different Counterfactual Scenarios (Reference: Men)

	Male	Female	Gender Gap	% Reduced	95% C.I.	
Observed	8.52	4.80	3.72			
**A** Decomposition 1 (8th grade)						
Characteristics effect: Changes after assigning to women the men’s 8th-grade S/E orientation and preparation		6.13	2.39	35.88	20.31	51.45
*Detailed decomposition*^a^						
S/E career expectations		[5.2, 5.6]	[2.9, 3.3]	[ 11.4, 21.4]		
Math and science interest		[4.1, 5.5]	[3.1, 4.4]	[−18.7, 17.6]		
Math, science, and reading performance		[4.8, 6.3]	[2.2, 3.8]	[−1.2, 41.0]		
Coefficient effect: Post–middle school choices		7.18	1.34	64.12	48.55	79.69
**B** Decomposition 2 (8th and 12th grades)						
Characteristics effect: Changes after assigning to women the men’s 12th-grade S/E orientation and preparation		7.65	0.87	76.71	58.65	94.77
*Detailed decomposition*^a^						
Plans to major in S/E		[6.3, 7.3]	[1.2, 2.2]	[41.1, 68.4]		
Math and science interest		[4.8, 5.2]	[3.3, 3.7]	[1.2, 11.8]		
Math, science, and reading performance		[5.0, 6.3]	[2.2, 3.5]	[5.6, 40.1]		
8th-and 12th-grade S/E orientation and preparation		7.84	0.68	81.75	62.74	100.77
Coefficient effect: Post–high school choices		5.48	3.04	18.25	−0.77	37.26

*Note:* The decomposition results presented in this table use the male coefficients as the reference coefficients. The corresponding analysis with female as the reference are presented in Table B1.

a The results from the detailed decomposition depend on the ordering of the variables, which reflect different causal models of educational decisions. The table presents the range of results indicated by the notation [*x, y*], which corresponds to the minimum and maximum contributions from the range of possible causal orderings.

## Footnotes

1 The relevance of the reference category is inherent in the decomposition method and explains the finding that a smaller proportion of the gap can be explained by eighthgrade characteristics when we use the female compared to the male coefficients as the reference. In particular, the differences between the groups is weighted either by the male or by the female coefficients. If the female coefficients for the covariates that have very different means for males and females are smaller than the male coefficients, the percentage explained by covariates will be smaller when females are used as the reference category. (For more details on the decomposition method, see Appendix B.)
