# Supplementary material for: Pathways to Science and Engineering Bachelor’s Degrees for Men and Women
Source: Sociol Sci. Author manuscript; Available in PMC 2014 Jul 31. (PMC4116814; doi:10.15195/v1.a4)
Supplement: Supplement [file NIHMS583664-supplement-Supplement.pdf]

sociological science

Supplement to:

Legewie, Joscha, and Thomas A. DiPrete. 2014. “Pathways to Science and Engineering Bachelor’s Degrees for Men and Women.” Sociological Science 1: 41-48.

Data and Coding of Variables

The analyses presented in this article are based on the NELS 1988 to 2000, which is a nationally representative sample of about 25,000 eighth-grade students who were first surveyed in spring 1988. Subsamples of these students were resurveyed in 1990, 1992, 1994, and 2000. We restrict the NELS 1988 to 2000 sample to students who participated in the 8th- and 12-grade surveys, and the 2000 follow-up, and exclude high school dropouts. As reported in the article, the size of this restricted sample is 10,230. Out of these cases, 3,140 (30.7 percent) had missing information on at least one of the variables (mostly, 12th-grade test scores). We use multiple imputations based on the chained-equations approach to recover missing values. Auxiliary variables such as 10th-grade test scores are used to improve the imputation mainly for the cases with missing 12th-grade test scores. Essentially the same results were obtained using casewise deletion. Note that all sample sizes are rounded to the nearest 10 (data contract requirement).

The coding of the variables is described in Table A1. The coding of the S/E variables (planned major in 12th grade and major of bachelor’s degree) separates science and engineering fields that are traditionally gender typed and usually require precollege preparation in math and science from other fields. On the basis of this general motivation, we exclude nursing and other health care majors from S/E fields. Sensitivity analysis that includes clinical and health sciences such as nursing in the S/E category showed essentially the same results, with small differences in the point estimates.

Field of Study Classifications

Non-S/E fields. No bachelor’s degree earned, agricultural business/production, agriculture/animal /plant sci, conservation/natural resources, forestry, architect/environmental design, graphic/industrial design, drama, speech, film arts, music, fine arts/art history, fpa: other, accounting, finance, ops research/administrative science, business admin /management, hrd/labor relations, other business, other business support, medical office support, marketing/distribution, journalism, communications, radio/TV/film, communication technologies, early childhood education, elementary education, secondary education, special education, physical education, education: other, med/vet lab tech/assist, dental assist/hygiene, hper, allied health: other, physical therapy, occupational therapy, other therapies, speech path/audiology, clinical health sci, nursing, health/hospital admin, public health, oth health sci/profess, para-legal/pre-law, law, psychology, anthropol/archaeology, economics, geography, history, sociology, political science, internat relations, other, amer studies/civiliz, area studies, ethnic studies, retailing, hospitality mgmnt, real estate, information technols, other personal service, engin tech: el/electron, computer technology, foreign languages, nutrition/food sci, textiles/fashion, fcs and oth human ecology, child study/guidance, culinary arts/food mgt, english/amer literature, writing: creative/tech, letters: other, liberal/general studies, library/archival sci, women’s studies, environ studies, biopsychology, integrated/gen science, interdisc humanities, social sci: general, interior design, recreation/sports, philosophy, religious studies, theology, Bible studies, clin/counsel psych, admin of justice, social work, public adminis-

Table A1: Description of Variables

Variable	Description
Occupational expectations (8th grade)	Coding based on the survey question "What kind of work do you expect to be doing when you are 30 years old?" The question provides thirteen answer categories, such as craftspersons, housewife, and business owner. Eighth-grade expectations for a career in S/E are defined based on the answer category "science or engineering professional, such as engineer or scientist."
S/E major plans (12th grade)	Our coding is based on two questions from the NELS 1994 second follow-up survey. These questions are the filter question "Do you plan to continue your education past high school at some time in the future?" and the intended field of study question "Indicate the field that comes closest to what you would most like to study if you go to school." We categorize the responses into two groups: (1) no college or college without S/E major and (2) college with plans to major in a S/E field. The first category includes students who do not intend to study at a four-year college and those who intend to study agriculture, architecture, art, business, communications, education, English, ethnic studies, foreign language, health occupations, home economics, interdisciplinary studies, music, philosophy/religion, preprofessional, psychology, social sciences, and other fields. The second category includes students who intend to study in S/E fields, which are defined as biological science, computer science, engineering, mathematics, and physical science.
Bachelor's degree in S/E field	Attainment of a bachelor's degree in S/E fields is defined as graduating from a four-year college in a S/E field until 2000 (eight years after the normal high school graduation). S/E field follows the same definition as the planned major in 12th grade. The coding of S/E fields is based on the recoded variable "majcod4" from the Postsecondary Education Transcript Study (PETS)—a NELS supplement. This variable combines information from the fourth follow-up survey with postsecondary transcript data to obtain detailed field codes for bachelor's degrees. See Appendix A for a detailed description of the field of study codes.
Performance (8th and 12th grade)	Eighth and 12th-grade reading, math, and science test scores (separate, cts variables)
Math and science interest (8th grade)	Four variables with 4-point Likert scale:<br>"I usually look forward to mathematics class"<br>"I usually look forward to science class"<br>"Math will be useful in my future"<br>"Science will be useful in my future"
Math and science interest (12th grade)	Measured based on two derived variables from the NELS PETS supplement, mainly based on questions about the reasons why a student took certain classes.

tration, human/community serv, graphic/print communic, and air transport.

S/E fields. Biochemistry, biological science: other, math sciences/statistics, chemistry, geology/earth science, physics, phys sci: other, computer programming, data/information management, computer science, electrical/communication engineer, chemical engineering, civil engineering, mechanical engineering, engineering: other, computer engineering, and engineering tech: nonelect.

Counterfactual Decomposition

We use Blinder–Oaxaca decomposition techniques for nonlinear models (Fairlie 2005) to analyze the gender gap in S/E bachelor's degree attainment by gender and determine the contribution of different life-course periods. Blinder–Oaxaca decomposition is a popular approach to study wage gaps by race and gender. It divides the differences between two groups into the part that is explained by a set of observed characteristics, such as educa-

tion and work experience (the endowment effect), and an unexplained part related to residual differences that are often interpreted as discrimination but also subsume group differences in unobserved characteristics (the coefficient effect). Here we use this counterfactual decomposition technique to divide the gender gap in S/E bachelor's degree attainment in the part that is explained by gender differences in 8th- and 12th-grade orientation toward and preparation for S/E (endowment effect) and the unexplained part. The unexplained portion of the gap is generally difficult to interpret but can be understood as an upper-bound estimate for the role of post–middle school (first decomposition) and post–high school (second decomposition) choices and transitions.[1] This approach allows us to estimate the gender gap under two counterfactual scenarios: "how would the gender gap in S/E degrees change if women had the same orientation toward and preparation for S/E in middle school (decomposition 1) and at the end of high school (decomposition 2)?" Following Fairlie (2005), the decomposition of the gender gap in S/E bachelor's degree attainment can be expressed as

$$\overline{Y}^M - \overline{Y}^W = \left[\sum_{i=1}^{N^M} \frac{\Phi(\mathbf{X}^M \hat{\beta}^M)}{N^M} - \sum_{i=1}^{N^W} \frac{\Phi(\mathbf{X}^W \hat{\beta}^M)}{N^W}\right] + \left[\sum_{i=1}^{N^W} \frac{\Phi(\mathbf{X}^W \hat{\beta}^M)}{N^W} - \sum_{i=1}^{N^W} \frac{\Phi(\mathbf{X}^W \hat{\beta}^W)}{N^W}\right],$$

where Y is an $n \times 1$ vector for the dependent variable so that $\overline{Y}^M$ denotes the proportion of men with a S/E bachelor's degree, $\mathbf{X}$ is an $n \times k$ matrix of independent variables, β is a $k \times 1$ vector of coefficients from a regression $P(Y) = logit^{-1}(X\beta)$, and Φ an inverse-logit function with $f(x) = e^x/(1+e^x)$. The superscripts M and W index gender-specific vectors and matrices.

[1] The estimates for 8th- and 12th-grade characteristics are lower bound, and for post–middle and high school choices, they are upper bound, because unobserved factors are subsumed in the second part. In contrast to research on discrimination, this nature of the estimates further supports our main argument that the gender gap is largely explained by gender differences at the end of high school. In the end, however, we believe that our set of variables is comprehensive and captures most important characteristics considering that the variables are directly related to the selection of students in schools, which is important from a causal perspective (Legewie 2012).

The first term on the right-hand side corresponds to the part of the gap that can be attributed to differences in observed characteristics (endowment effect), and the second term corresponds to the unexplained part related to differences in coefficients (coefficient effect). The contribution of each variable can be determined by assigning women the men's distribution of that variable while leaving the distributions of the other variables unchanged (Fairlie 2005). Assuming that $N_W = N_M$, the contribution of x_1 can be expressed as

$$\frac{1}{N^W} \sum_{i=1}^{N^W} \left[\Phi\left(\hat{\alpha}^* + x_{1i}^M \hat{\beta}_1^* + x_{2i}^M \hat{\beta}_2^*\right) - \Phi\left(\hat{\alpha}^* + x_{1i}^W \hat{\beta}_1^* + x_{2i}^M \hat{\beta}_2^*\right) \right], \quad (1)$$

where $\hat{\beta}^*$ denotes the logistic regression coefficients from the pooled sample. This approach relies on the assumption that the two groups are equally large (Fairlie 2005). To circumvent this assumption, we average over multiple computations of the decomposition based on 1,000 equally large samples from the two groups.

The results of this decomposition analysis are sensitive to the reference coefficients and the ordering of the covariates. The formulas use the male coefficients as the reference coefficients, but the same analyses with women as the reference category are presented in Table B1 and discussed throughout the article. In one case, the effect of differences in coefficients is weighted, and in the other case it is weighted by the female coefficients. If the female coefficients for the covariates that have very different means for males and females are smaller than the male coefficients, then the percentage explained by covariates will be smaller when females are used as the reference category.

The ordering effect only matters for the detailed decomposition and reflects different underlying causal models of educational decisions. As discussed in the main text of the article, we report ranges of estimates relating to different assumptions about the causal order of the covariates.

Additional Analyses

We conducted the same counterfactual decomposition with women as the reference group, for

different S/E subfields and for the subset of students who graduate from college with a four-year bachelor's degree. First, Table B1 shows the result for women at the reference, which are discussed throughout the main text of the article. Second, Table C1 presents the result for physical science and engineering (PS/E), which includes math and computer science and excludes biological and life science. As such, PS/E represents the fields for which women have made relatively few gains, as compared to biological and life science, in which they have largely caught up and partly surpassed men. Finally, Table C2 presents the results conditional on graduating from a four-year college so that it examines gender differences in the majority of students who actually obtain a bachelor's degree. The findings from both additional analyses show small differences but are largely comparable to the ones presented in the main paper. They further support the argument that the gender gap would be reduced substantially if men and women had the same orientation toward and preparation for science and engineering at the end of high school.

References

Fairlie, R. W. 2005. "An Extension of the Blinder–Oaxaca Decomposition Technique to Logit and Probit Models." *Journal of Economic and Social Measurement* 30:305–16.

Table A2: Comparison of the Observed Gender Gap in Science and Engineering Degrees with the Gap under Different Counterfactual Scenarios (Reference: Women)

	Male	Female	Gender Gap	% Reduced	95% C.I.	
Observed	8.52	4.80	−3.72			
**A** Decomposition 1 (8th grade)						
Characteristics effect: Changes after assigning to women the men's 8th-grade S/E orientation and preparation	7.45		−2.65	28.92	45.30	12.53
Detailed decomposition[a]						
S/E career expectations	[8.3, 8.4]		[−3.6, −3.5]	[4, 6.1]		
Math and science interest	[7.7, 9]		[−4.2, −2.9]	[−12.8, 21.4]		
Math, science, and reading performance	[7.1, 8.5]		[−3.7, −2.3]	[1.5, 37.7]		
Coefficient effect: Post–middle school choices	5.87		−1.07	71.08	54.70	87.47
**B** Decomposition 2 (8th and 12th grades)						
Characteristics effect: Changes after assigning to women the men's 12th-grade S/E orientation and preparation	5.78		−0.99	73.59	97.58	49.59
Detailed decomposition[a]						
Plans to major in S/E	[6.3, 7.1]		[−2.3, −1.6]	[38.1, 58.4]		
Math and science interest	[8.1, 8.9]		[−4.1, −3.3]	[−9.5, 12.5]		
Math, science, and reading performance	[7.2, 8.1]		[−3.3, −2.4]	[10.2, 36.9]		
8th- and 12th-grade S/E orientation and preparation	5.26		−0.46	87.69	114.85	60.53
Coefficient effect: Post–high school choices	8.06		−3.26	12.31	−14.85	39.47

Note: The decomposition results presented in this table use the female coefficients as the reference coefficients. The corresponding analysis with male as the reference are presented in Table 1.

[a]The results from the detailed decomposition depend on the ordering of the variables, which reflect different causal models of educational decisions. The table presents the range of results indicated by the notation $[x, y]$, which corresponds to the minimum and maximum contributions from the range of possible causal orderings.

Table A3: Comparison of the Observed Gender Gap in Physical Science and Engineering Degrees with the Gap under Different Counterfactual Scenarios (Reference: Men)

	Male	Female	Gender Gap	% Reduced	95% C.I.	
Observed	6.64	2.20	4.44			
**A** Decomposition 1 (8th grade)						
Characteristics effect: Changes after assigning to women the men's 8th-grade S/E orientation and preparation		3.26	3.38	23.94	11.97	35.92
Detailed decomposition[a]						
S/E career expectations		[2.5, 2.7]	[3.9, 4.2]	[6.3, 12.3]		
Math and science interest		[1.4, 2.8]	[3.8, 5.2]	[−17.4, 14.6]		
Math, science, and reading performance		[2.1, 3.7]	[3, 4.5]	[−1.2, 32.8]		
Coefficient effect: Post–middle school choices		5.58	1.06	76.06	64.08	88.03
**B** Decomposition 2 (8th and 12th grades)						
Characteristics effect: Changes after assigning to women the men's 12th-grade S/E orientation and preparation		5.39	1.25	71.88	57.40	86.37
Detailed decomposition[a]						
Plans to major in S/E		[4.5, 6.1]	[0.6, 2.1]	[52.6, 86.9]		
Math and science interest		[2.2, 2.4]	[4.2, 4.4]	[0.8, 4.7]		
Math, science, and reading performance		[1.7, 3.2]	[3.5, 5]	[−12.3, 22.3]		
8th- and 12th-grade S/E orientation and preparation		5.42	1.22	72.48	58.03	86.93
Coefficient effect: Post–high school choices		3.42	3.22	27.52	13.07	41.97

Note: The decomposition results presented in this table use the male coefficients as the reference coefficients. The corresponding analysis with female as the reference are presented in Table A1.

[a]The results from the detailed decomposition depend on the ordering of the variables, which reflect different causal models of educational decisions. The table presents the range of results indicated by the notation $[x, y]$, which corresponds to the minimum and maximum contributions from the range of possible causal orderings.

Table A4: Comparison of the Observed Gender Gap in Science and Engineering Degrees with the Gap under Different Counterfactual Scenarios Conditional on Graduating from Four-Year College with a BA Degree (Reference: Men)

	Male	Female	Gender Gap	% Reduced	95% C.I.	
Observed	25.99	11.80	14.18			
**A** Decomposition 1 (8th grade)						
Characteristics effect: Changes after assigning to women the men's 8th-grade S/E orientation and preparation		16.18	9.80	30.89	19.46	42.32
Detailed decomposition[a]						
S/E career expectations		[13.3, 13.7]	[12.3, 12.6]	[10.9, 13.1]		
Math and science interest		[11.6, 13]	[13, 14.4]	[-1.4, 8.2]		
Math, science, and reading performance		[13.3, 14.7]	[11.2, 12.6]	[10.9, 20.7]		
Coefficient effect: Post–middle school choices		21.61	4.38	69.11	57.68	80.54
**B** Decomposition 2 (8th and 12th grades)						
Characteristics effect: Changes after assigning to women the men's 12th-grade S/E orientation and preparation		20.13	5.85	58.76	46.23	71.30
Detailed decomposition[a]						
Plans to major in S/E		[18.1, 18.9]	[7.1, 7.9]	[44.2, 49.7]		
Math and science interest		[12.3, 12.4]	[13.6, 13.7]	[3.3, 4.2]		
Math, science, and reading performance		[13.1, 13.9]	[12.1, 12.9]	[9.1, 14.8]		
8th- and 12th-grade S/E orientation and preparation		21.74	4.24	70.11	57.56	82.66
Coefficient effect: Post–high school choices		16.05	9.94	29.89	17.34	42.44

[a]The results from the detailed decomposition depend on the ordering of the variables, which reflect different causal models of educational decisions. The table presents the range of results indicated by the notation $[x, y]$, which corresponds to the minimum and maximum contributions from the range of possible causal orderings.
